# Differentiation of adipose-derived stem cells into functional chondrocytes by a small molecule that induces Sox9

**DOI:** 10.1038/s12276-020-0424-y

**Published:** 2020-04-21

**Authors:** Jiyun Lee, Chang Youn Lee, Jun-Hee Park, Hyang-Hee Seo, Sunhye Shin, Byeong-Wook Song, Il-Kwon Kim, Sang Woo Kim, Seahyoung Lee, Jong-Chul Park, Soyeon Lim, Ki-Chul Hwang

**Affiliations:** 1 Brain Korea 21 PLUS Project for Medical Science, Yonsei University, Seoul, Republic of Korea; 2 Institute for Bio-Medical Convergence, College of Medicine, Catholic Kwandong University, Gangneung, Republic of Korea; 3 Department of Integrated Omics for Biomedical Sciences, Yonsei University, Seoul, Republic of Korea; 4 Department of Medical Engineering, Yonsei University College of Medicine, Seoul, 03722 Republic of Korea

**Keywords:** Stem-cell differentiation, Osteoarthritis

## Abstract

Osteoarthritis (OA) is a common joint disease that results from the disintegration of joint cartilage and the underlying bone. Because cartilage and chondrocytes lack the ability to self-regenerate, efforts have been made to utilize stem cells to treat OA. Although various methods have been used to differentiate stem cells into functional chondrocytes, the currently available methods cannot induce stem cells to undergo differentiation into chondrocyte-like cells without inducing characteristics of hypertrophic chondrocytes, which finally lead to cartilage disintegration and calcification. Therefore, an optimized method to differentiate stem cells into chondrocytes that do not display undesired phenotypes is needed. This study focused on differentiating adipose-derived stem cells (ASCs) into functional chondrocytes using a small molecule that regulated the expression of Sox9 as a key factor in cartilage development and then explored its ability to treat OA. We selected ellipticine (ELPC), which induces chondrocyte differentiation of ASCs, using a GFP-Sox9 promoter vector screening system. An *in vivo* study was performed to confirm the recovery rate of cartilage regeneration with ASC differentiation into chondrocytes by ELPC in a collagenase-induced animal model of OA. Taken together, these data indicate that ellipticine induces ASCs to differentiate into mature chondrocytes without hypertrophic chondrocytes *in vitro and in vivo*, thus overcoming a problem encountered in previous studies. These results indicate that ELPC is a novel chondrocyte differentiation-inducing drug that shows potential as a cell therapy for OA.

## Osteoarthritis: Drug-induced stem cell differentiation shows therapeutic promise

A novel method of generating healthy cartilage cells from stem cells could repair and regenerate joints and bones damaged by osteoarthritis. The cells that produce and maintain cartilage tissue are known as chondrocytes. Stem cell therapy could potentially re-start cartilage and bone regeneration in patients with osteoarthritis, however scientists have struggled to successfully induce stem cells to differentiate into functioning chondrocytes. A new method developed by Soyeon Lim and Ki-Chul Hwang at the Catholic Kwandong University, Gangneung, South Korea, and co-workers uses a drug called ellipticine to boost the expression of Sox-9, a key protein involved in cartilage development. The researchers found that the ellipticine-based method induced a mature, healthy chondrocyte population in cell cultures and rat models of osteoarthritis. Rats injected with these cells recovered from cartilage damage, suggesting a potential osteoarthritis therapy.

## Introduction

Osteoarthritis (OA) is a major degenerative and common chronic joint disease caused mainly by aging^1,2^. Moreover, OA has been observed in younger populations due to systemic and genetic factors^3–5^. The articular cartilage composed of chondrocytes and a dense extracellular matrix (ECM) is a key component of joints that have a limited capacity for intrinsic healing and repair^6,7^. During the progression of OA, active ECM remodeling occurs with environmental changes such as severe or chronic inflammation, which in turn leads to the induction of the maturation of hypertrophic chondrocytes. Hypertrophic chondrocytes characteristically overexpress hypertrophic markers, runt-related transcription factor 2 (RUNX2), Type X collagen, Indian hedgehog, and Transglutaminase-2. Hypertrophic chondrocytes are also known to synthesize various matrix-degrading enzymes, such as matrix metalloproteinase 13 (MMP13) and a disintegrin and metalloproteinase containing thrombospondin motifs (ADAMTS4, 5), which accelerate the decomposition of the ECM, leading to cartilage loss and calcification^8,9^

Although various methods from alternative medicine to surgical procedures have been utilized for OA patients, there is no way to reverse the onset of this disease. The most recent therapeutic approaches for OA involve the use of stem cells^10^. Adipose-derived stem cells (ASCs) have recently been widely used because they are easier to obtain than bone marrow-derived stem cells^11,12^. However, previous studies have demonstrated that hypertrophic chondrocytes are a major problem in differentiated chondrocytes using stem cells ^13,14^.

To regulate the expression of critical genes and proteins involved in stem cell differentiation, previous studies have used systems such as viral vectors, microRNAs, siRNAs, and shRNAs. However, conventional systems have limitations regarding cytotoxicity, delivery, and maintenance^15–17^. Therefore, we used small molecules that are stable and transferable in the cells to control the expression of genes and proteins. Consequently, a new small molecule-based method was used to screen for small molecule drugs that could induce the differentiation from ASCs into chondrocytes. Ultimately, the goal is to induce the optimal differentiation of ASCs into mature chondrocytes using a new small molecule-based method.

## Materials and methods

### Materials

Ellipticine (ELPC, 5,11-dimethyl-6H-pyrido[4,3-b]carbazole) was purchased from Enzo Life Science, Inc. In–house chemical libraries, receptor agonists and antagonist kinase inhibitors, and ion channel activators and inhibitors were used^18^. Dulbecco’s modified Eagle’s medium (DMEM), fetal bovine serum (FBS), and penicillin-streptomycin were obtained from the same corporation (Life Technologies, Grand Island, NY, USA). For polymerase chain reaction (PCR), oligonucleotides were synthesized by Bioneer (Bioneer, Daejeon, South Korea), and RNA was extracted using chloroform and 2-propanol (Sigma-Aldrich, St. Louis, MO, USA). Reverse transcription for cDNA synthesis was conducted using a reverse transcription system (Promega Corporation, Madison, WI, USA), and PCR was performed with Ex Taq, dNTP mixture (2.5 mM each), and 10x Ex Taq buffer (TaKaRa Bio, Inc., Otsu, Japan). Western blot and immunofluorescence antibodies were used to detect type II collagen, aggrecan, type X collagen, p53, p-p53, and β-actin (Santa Cruz Biotechnology, USA). Secondary antibody usage was divided into 2 types: mouse or rabbit (Enzo Life Sciences, Inc., Farmingdale, NY, USA) for western blot analysis (horseradish peroxidase (HRP)-conjugated) and mouse, rabbit, or goat (Jackson Immunoresearch Laboratories, West Grove, PA, USA; Vector Laboratories, Burlingame, CA, USA; Abcam, USA) for immunostaining (fluorescein isothiocyanate, FITC; phycoerythrin, PE; or allophycocyanin, APC-conjugated). Western blotting detection systems were obtained from GE Healthcare Life Sciences (Uppsala, Sweden), and 4′,6-diamidino-2-phenylindole was obtained from Life Technologies (USA).

### Sox9 promoter gene assay

The promoter region for the sex-determining region Y-type high mobility group box (Sox)9 (−1034 bp to +67 bp) used in this study was based on the promoter region described in DC Colter et al.^19^. The PCR product of the promoter region was cloned into the pAcGFP1-1 vector. The conjugated Sox9 promoter GFP vector was transfected into ASCs using the TransIT-X2 Dynamic Delivery system. After transfection, the GFP intensity was examined under fluorescence microscopy (Glomax® Explorer multimode microplate reader, Promega, WI, USA).

### Animals

Adult 8-week-old Sprague-Dawley male rats were used (Koatech, Pyeongtaek, Korea) from Harlan USA. All animal experimental procedures were approved by the Institutional Animal Care and Use Committee, Catholic Kwandong University College of Medicine, and the Association for Assessment and Accreditation of Laboratory Animal Care and were performed in accordance with the Guidelines and Regulations for Animal Care.

### Adipose-derived stem cell culture

Human ASCs were purchased from Invitrogen (CA, USA) and cultured in growth media (DMEM supplemented with 10% FBS, 100 unit/ml penicillin, and 100 μg/ml streptomycin) at 37 °C and 5% CO_2_. Experiments used cells between passages 3 and 7 for differentiation.

### Pellet culture system

ASCs were cultured by an improved pellet culture system described by Johnstone et al.^20^ When cartilage formation proceeds in a stable and smooth manner, the pellet will maintain a rounded shape while it grows. ASCs (2.5 × 10^5^) were centrifuged at 600×*g* for 5 min in a 15-ml polypropylene tube. The resulting pellets were treated with an ELPC final concentration of 1 μM in 10% FBS DMEM for 16 days. The medium and the ELPC were replaced with fresh medium and ELPC once every 3 days.

### Cell viability assay

ASCs were plated in 96-well cell culture plates in triplicate at 5 × 10^3^ cells/well. After treatment with ELPC for 24 h or 48 h in ASCs, EZ-Cytox reagent (DoGEN, Seoul, Korea) was added to each well and incubated at 37 °C for 2 h to react. The sample absorbance was measured using a microplate reader (Thermo Fisher Scientific, MA, USA) at 450 nm.

### Reverse transcription-polymerase chain reaction (RT-PCR)

Total RNA was extracted using TRIzol. Chloroform was added to separate each sample into layers of RNA, DNA, and proteins, and then, each sample was centrifuged at 12,000 rpm and 4 °C for 15 min. Next, the RNA from each sample was collected in a new tube, and 2-propanol was added to obtain the pellet, after which the centrifugation was repeated at 12,000 rpm at 4 °C for 10 min. The pellet was washed in 75% (v/v) ethanol and mixed with diethylpyrocarbonate (DEPC) dissolved in water. After centrifugation at approximately 12000 rpm at 4 °C for 5 min, the pellet was dried at room temperature. Finally, the pellet was dissolved in 30 μL of nuclease-free water (NFW). The quality and quantity of RNA were estimated by calculation of OD260/OD280 ratios using a spectrophotometer. Complementary DNA (cDNA) was synthesized using the RT System kit. The RNA was added to the oligo dT primer, dNTP mixture, RTase, RNase inhibitor, and buffer. The generated cDNA was mixed with each primer, dNTP mixture, Taq polymerase, and reaction buffer in the PCR tube. PCR conditions consisted of denaturation at 94 °C for 3 min, followed by 30 cycles each featuring denaturation at 94 °C for 30 s, annealing at 48–60 °C for 30 s, and elongation at 72 °C for 30 s, and then, the reaction was maintained at 72 °C for 10 min. The following primer sequences were used: F: GAGGAAGTCGGTGAAGAACG and R: ATCGAAGGTCTCGATGTTGG for Sox9; F: TGAGGAGGGCTGGAACAAGT and R: GGAGGTGGTAATTGCAGGGA for Aggrecan; F: TGGAGAAACCATCAATGGTGG and R: TGGAGAAACCATCAATGGTGG for Type II collagen; F: ATGACCCAAGGACTGGAATCTTTA and R: ATGACCCAAGGACTGGAATCTTTA for Type X collagen; F: AAGGGTCCACTCTGGCTTTG and R: CTAGGCGCATTTCAGGTGCT for RUNX2; F: TTTCCCTGGCAAGGACTATG and R: GGAGGAGAACTGGACACCAC for ADAMTS4; F: TGACCATGAGGAGCACTACG and R: TGGGAGAGGCCAAGTAAATG for ADAMTS5; F: GTGGTGTGGGAAGTATCATCA and R: GCATCTGGAGTAACCGTATTG for MMP13; F: GAAACTACTTCCTGAAAACAACGT and R: GCCTCACAACCTCCGTACT for p53; F: CATGGGTGTGAACCATGAGA and R: GGTCATGAGTCCTTCCACGA for GAPDH. PCR products were separated by electrophoresis on 1.2% (w/v) agarose gels. Gel-Doc was used to visualize the bands.

### Nuclear extraction

The nuclei and cytoplasm of ASCs were extracted using NE-PER nuclear and cytoplasmic extraction reagents kits (Thermo Scientific, IL, USA). ASCs were harvested with trypsin-EDTA and centrifuged at 500 × *g* for 5 min to collect the cell pellet. Then, the cell pellet was washed with PBS, and 1–10 × 10^6^ cells were transferred to a 1.5-ml microcentrifuge tube and pelleted by centrifugation at 500 × *g* for 3 min. After the PBS was removed, cytoplasmic extraction reagent I (CER I) was added to the cell pellet. The cell pellet was vortexed to fully suspend it on the highest setting for 15 s and then incubated on ice for 10 min. Then, cytoplasmic extraction reagent II (CER II) was added to the sample, vortexed for 5 s, placed on ice for 1 min, vortexed for 5 s, and centrifuged for 5 min at maximum speed in a microcentrifuge (~16,000 × *g*). The supernatant was immediately transferred to a new tube and stored on ice until use. The supernatant contained cytoplasmic proteins, and the pellet contained the nuclear fraction. After addition of ice-cold nuclear extraction reagent (NER) to the pellet, vortexing for 15 s and incubation on ice for 10 min was repeated four times. Then, the sample was centrifuged at maximum speed in a microcentrifuge (~16,000 × *g*) for 10 min, and the supernatant fraction was transferred to a new tube. The supernatant included the nuclear extract.

### Western blot analysis

The cells were washed with PBS and lysed in lysis buffer with proteinase and phosphatase inhibitors. Protein concentrations were determined using the BCA Protein Assay Kit. Then, the same concentration of proteins was separated on a sodium dodecyl sulfate-polyacrylamide gel and transferred to a polyvinylidene difluoride membrane. After the membrane was blocked in 5% skim milk mixed with 0.1% Tween 20 in TBS buffer for 1 h at room temperature, it was rinsed twice with TBS buffer and incubated with primary antibody overnight at 4 °C. Next, the membrane was washed three times with 0.1% Tween 20 TBS buffer for 10 min and incubated for 1 h at room temperature with HRP-conjugated secondary antibodies. After extensive washing, the bands were detected with an enhanced chemiluminescence reagent. The band intensities were quantified using NIH ImageJ version 1.34e software.

### ADAMTS4 and ADAMTS5 ELISA verification

The protein activities of ADAMTS4 and ADAMTS5, which are released as active protease forms in the cell culture supernatant, were measured using ADAMTS4 and ADAMTS5 ELISA kits (R&D Systems). The ADAMTS4 and ADAMTS5 capture antibodies were coated in a 96-well microplate overnight at room temperature. After three washes, blocking with reagent diluent was performed for 1 h, and the standard and sample were added and reacted at room temperature for 2 h. The plate was washed three times, incubated with detection antibody for 2 h, and washed again. Streptavidin-HRP was added and reacted for 20 min in the dark. After the substrate solution was added for 20 min, the reaction was stopped using stop solution, and the value was read at 450 nm using a microplate reader.

### Transcription factor activation array

Transcription factor activation was measured using a TF activation profiling plate array and customized array (Signosis, Inc., CA, USA). The nuclei of ASCs were extracted using a Nuclear Extraction Kit (Signosis, Inc.). ASCs were washed with PBS, and then, the Buffer I working reagent mixture was added for 10 min. The ASCs were removed from the plate using a scraper, transferred to a microcentrifuge tube and centrifuged at 12000 rpm for 5 min. The supernatant was discarded completely, and the Buffer II working reagent mixture was added for 2 h. The sample was centrifuged at 12,000 rpm for 5 min, and then, the supernatant was transferred to a new tube. For TF/DNA complex formation, the TF probe and nuclear fraction were mixed in a tube for 30 min. The TF/DNA complex was separated from the free probe and incubated in a hybridization plate overnight. Finally, transcription factor activity was detected using a luminometer.

### Osteoarthritis induction and ASC injection

Osteoarthritis was induced using 250 g male Sprague-Dawley (SD) rats. Rats were anesthetized by intraperitoneal administration of 20 mg/kg Zoletil and 5 mg/kg Rompun. Anesthesia was performed twice by intra-articular injection for 1 week, where 30 μl of either sterile saline (control group) and 250 U or 500 U of type II collagenase dissolved in saline and filtered through a 0.22-μm membrane was injected. ASCs induce differentiation into chondrocytes after treatment with ELPC for 16 days prior to injection. One week after the injection of Type II collagenase, normal ASCs and differentiated ASCs were injected with 1 × 10^6^ cells in PBS.

### Alcian blue staining

Alcian blue is a blue stain associated with the sulfated glycosaminoglycan of the cartilage matrix. After 16 days, the cells were rinsed once with PBS and fixed with 4% formaldehyde solution for 1 h at room temperature. Then, Alcian blue staining solution with 3% acetic acid solution (pH 2.5) was added to each sample for 30 min. After staining, the samples were washed in running tap water for 2 min and finally mounted with mounting solution.

### Safranin O staining

The knee joint paraffin sections were stained using a safranin O staining kit. First, the section was deparaffinized, rehydrated, rinsed with tap water and then stained with 0.1% fast green solution for 5 min. The sample was rinsed with 1% acetic acid for 10 s and then stained using 0.1% safranin O staining solution for 30 min. Finally, the section was dehydrated and mounted with mounting solution.

### Immunofluorescence

The knee joint was fixed overnight with 10% (v/v) formaldehyde. The knee joint was embedded in paraffin and transversely sectioned into serial thick sections. After deparaffinization, rehydration, and rinsing with tap water, sodium citrate antigen retrieval was performed using 10 mM sodium citrate (pH 6.0) in a microwave for 10 min. Sections were incubated in 1% H_2_O_2_ to quench endogenous peroxidase. The tissue sections were treated with 0.1% sodium borohydride to remove autofluorescence and were blocked in 2.5% normal horse serum. After blocking, the sections were incubated with primary antibody overnight. The sections were analyzed for DAPI, Aggrecan, and collagen type 2 and 10 by an LSM700 confocal laser scanning microscope.

### Statistical analysis

The results are expressed as the mean ± SD from at least three independent experiments. Statistical analyses were performed using Student’s *t*-test. Comparisons between more than two groups were performed by one-way ANOVA using Bonferroni’s correction. Relationships were considered statistically significant when the *p*-value was <0.05.

## Results

### Screening of small molecules that induce the differentiation of ASCs into chondrocytes

**Fig. 1:**
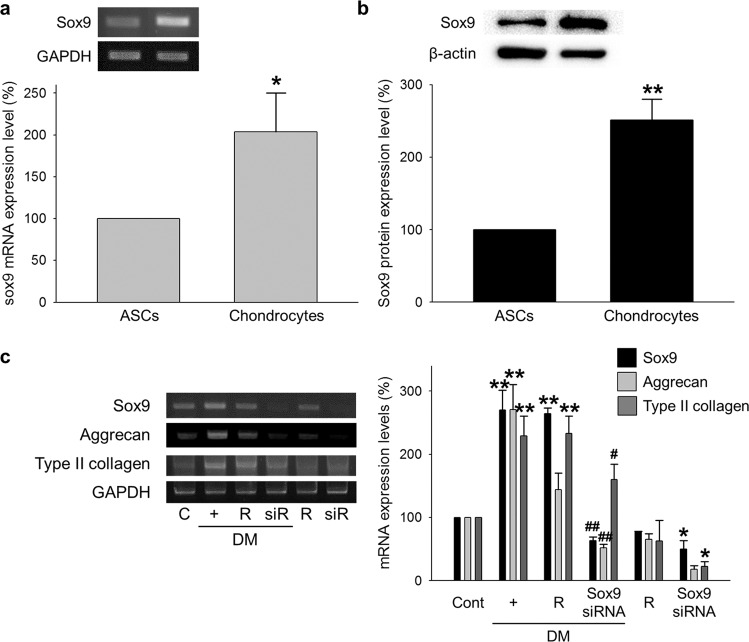
Importance of Sox9 in chondrogenic differentiation. **a**, **b** mRNA and protein expression of Sox9 in ASCs and chondrocytes. Normalized by GAPDH and B-actin. *p < 0.05 compared to ASCs **p < 0.001 compared to ASCs. *n* = 3. **c** ASCs were transfected with 50 nM Sox9 siRNA for 24 h and cultured in chondrocyte differentiation medium (DM). The cells were transfected with the Sox9 siRNA every 3 days for 16 days while cultured in DM. The mRNA expression of Sox9, Aggrecan, and Type II collagen in the Sox9 siRNA-transfected ASCs. C and Cont control, R reagent-transfected ASCs, siR Sox9 siRNA-transfected ASCs, DM chondrogenic differentiation induction medium. *p < 0.05, **p < 0.001 compared to the control, ^#^*p* < 0.05, ^##^*p* < 0.001 compared to the reagent-transfected and DM-treated ASCs. *n* = 3.

**Fig. 2:**
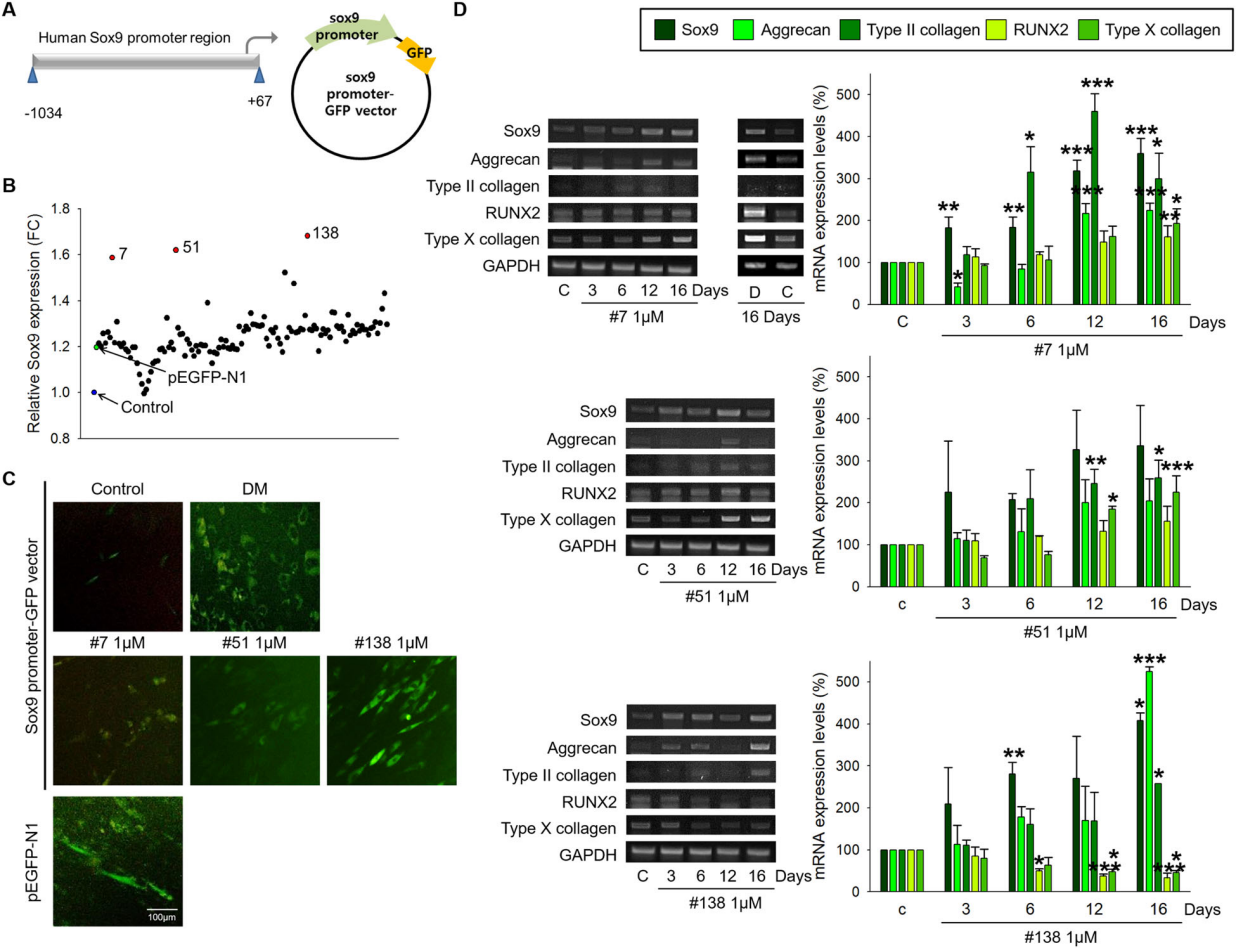
Chemical screening of the Sox9 inducer from a small molecule library. **a** Vector generation strategy containing the Sox9 promoter region and a GFP tag. **b** After the ASCs were transfected with the Sox9 promoter-GFP vector, 1 μM small molecules were added for 24 h. GFP expression was measured using a fluorescence microplate reader. **c** Selected candidate small molecules were added to ASCs transfected with the Sox9 promoter-GFP vector. GFP expression in the ASCs transfected with the Sox9 promoter-GFP vector was observed by fluorescence microscopy. A pEGFP-N1 vector was used as a positive control to evaluate the transfection efficiency. Chondrogenic differentiation medium (DM) was also used as a positive control to evaluate Sox9 expression. **d** ASCs were treated with 1 μM of each of the three selected candidate drugs every 3 days for 16 days. The expression of mature chondrocyte markers (Sox9, Aggrecan, and Type II collagen) and hypertrophic chondrocyte markers (RUNX2 and Type X collagen) was measured using PCR of the Drug 7-, Drug 51-, and Drug 138-treated ASCs. D DMSO-treated ASCs, *p < 0.01, **p < 0.05, ***p < 0.001 compared to the control. *n* = 3.

First, we found that the mRNA and protein levels of the sex-determining region Y-type high mobility group box (Sox)9 were higher in chondrocytes than in ASCs (Fig. 1a, b). Next, we confirmed an important role of Sox9 during the differentiation of ASCs into chondrocytes. The increased expression of Sox9, Aggrecan, and Type II collagen in the DM-treated ASCs was inhibited in the Sox9 siRNA-transfected ASCs (Fig. 1c). A specific vector system containing the Sox9 promoter region and GFP was used to screen for a drug that increases Sox9 expression (Fig. 2a). The relative Sox9 expression was analyzed based on the overexpression of GFP under the treatment of small molecules (Fig. 2b) and was confirmed using fluorescence microscopy (Fig. 2c). Finally, three candidate drugs were selected, and the differentiation capability of the drugs was determined by the mRNA expression levels of mature chondrocyte and hypertrophic chondrocyte markers. The expression levels of Sox9, Aggrecan, and Type II collagen were increased in the ASCs treated with each of the three drugs, whereas those of RUNX2 and Type X collagen were increased in the cells treated with drugs 7 and 51 and not in the ASCs treated with drug 138, as shown in Fig. 2d.

### ELPC induces chondrocyte differentiation in adipose-derived stem cells

**Fig. 3:**
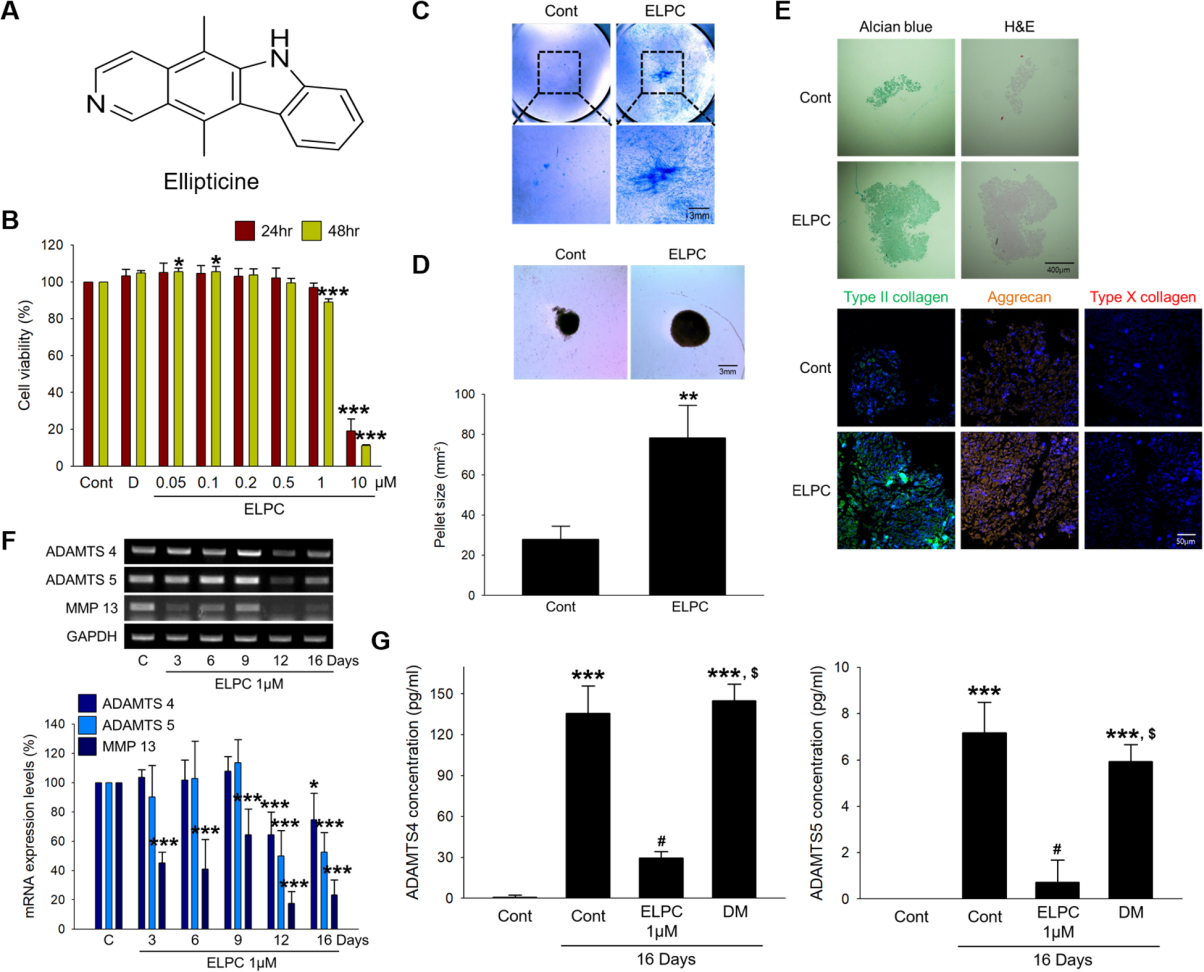
Ellipticine induces chondrogenic differentiation in ASCs. **a** Structure of ellipticine. **b** Concentration-dependent cell viability was measured by cell viability assays in ASCs. D DMSO-treated ASCs *p < 0.05, ***p < 0.001 compared to the control. *n* = 3. **c** The ability of ELPC to induce differentiation was confirmed using Alcian blue staining after ASCs were incubated with ELPC for 16 days. Scale bar: 3 mm. **d** A 3D pellet culture was performed using ASCs or ELPC-treated ASCs for 16 days. **p < 0.01 compared to the control. *n* = 3. **e** The untreated ASC and ELPC-treated ASC pellets were stained using Alcian blue and H&E. The expression of Type II collagen, Aggrecan, and Type X collagen was detected by immunofluorescence. Nuclei were stained with DAPI (Blue). Scale bar: upper 400 μm and lower 50 μm. **f** The mRNA expression of the extracellular matrix degradation enzymes ADAMTS4, ADAMTS5, and MMP13. Normalized by GAPDH. *p < 0.05, ***p < 0.001 compared to the control. *n* = 3. **g** The concentrations of secreted ADAMTS4 and ADAMTS5 were measured by ELISAs. ***p < 0.001 compared to the control, ^#^*p* < 0.001 compared to the 16-day control, ^$^*p* < 0.001 compared to the ELPC-treated ASCs. C and Cont control, DM chondrogenic differentiation induction medium, ELPC ELPC-treated ASCs. *n* = 3.

Drug 138, known as ellipticine (ELPC), is a natural tetracyclic compound (Fig. 3a). The cytotoxicity in ASCs prior to further experiments was investigated; 1 μM ELPC resulted in a slight reduction in cell viability, and 80% or more of the ASCs underwent cell death with 10 μM ELPC (Fig. 3b). To further confirm the ability of ELPC to induce the differentiation of ASCs into chondrocytes, we performed Alcian blue staining after the ASCs were incubated with ELPC for 16 days. Figure 3c shows that the ELPC-treated ASCs had more blue and condensed areas than the untreated ASCs. The ELPC-treated ASCs maintained more rounded pellets and a greater diameter and total size than the untreated ASCs (Fig. 3d). The expression levels of Aggrecan and Type II collagen were increased in the ELPC-treated ASC pellets, whereas the expression of Type X collagen was not increased (Fig. 3e). We also confirmed that the mRNA levels of ECM-degrading enzymes were decreased in a time-dependent manner by ELPC treatment (Fig. 3f). In addition, the levels of ADAMTS4 and ADAMTS5 were significantly decreased in the medium of the ELPC-treated ASCs (Fig. 3g).

### ELPC increases the expression of Sox9 by regulating p53 during chondrogenesis

**Fig. 4:**
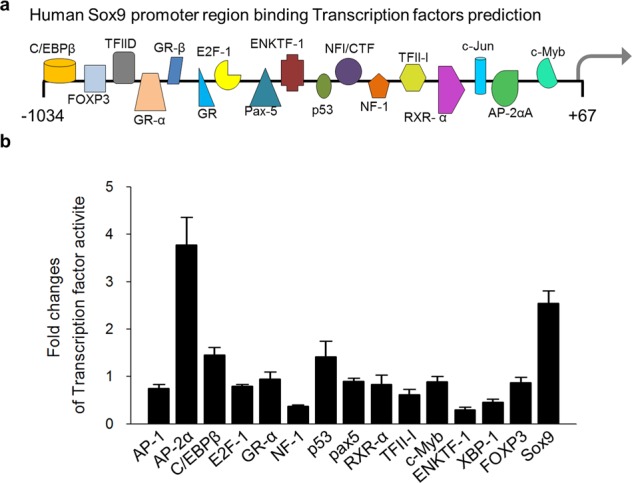
Transcription factors associated with ELPC in the Sox9 promoter region. **a** Transcription factors binding to the Sox9 promoter were predicted by the PROMO 3.0 program. **b** Transcription factor activity, predicted to bind a Sox9 promoter region, was measured using a TF activation plate array kit (customizing kit, Signosis).

**Fig. 5:**
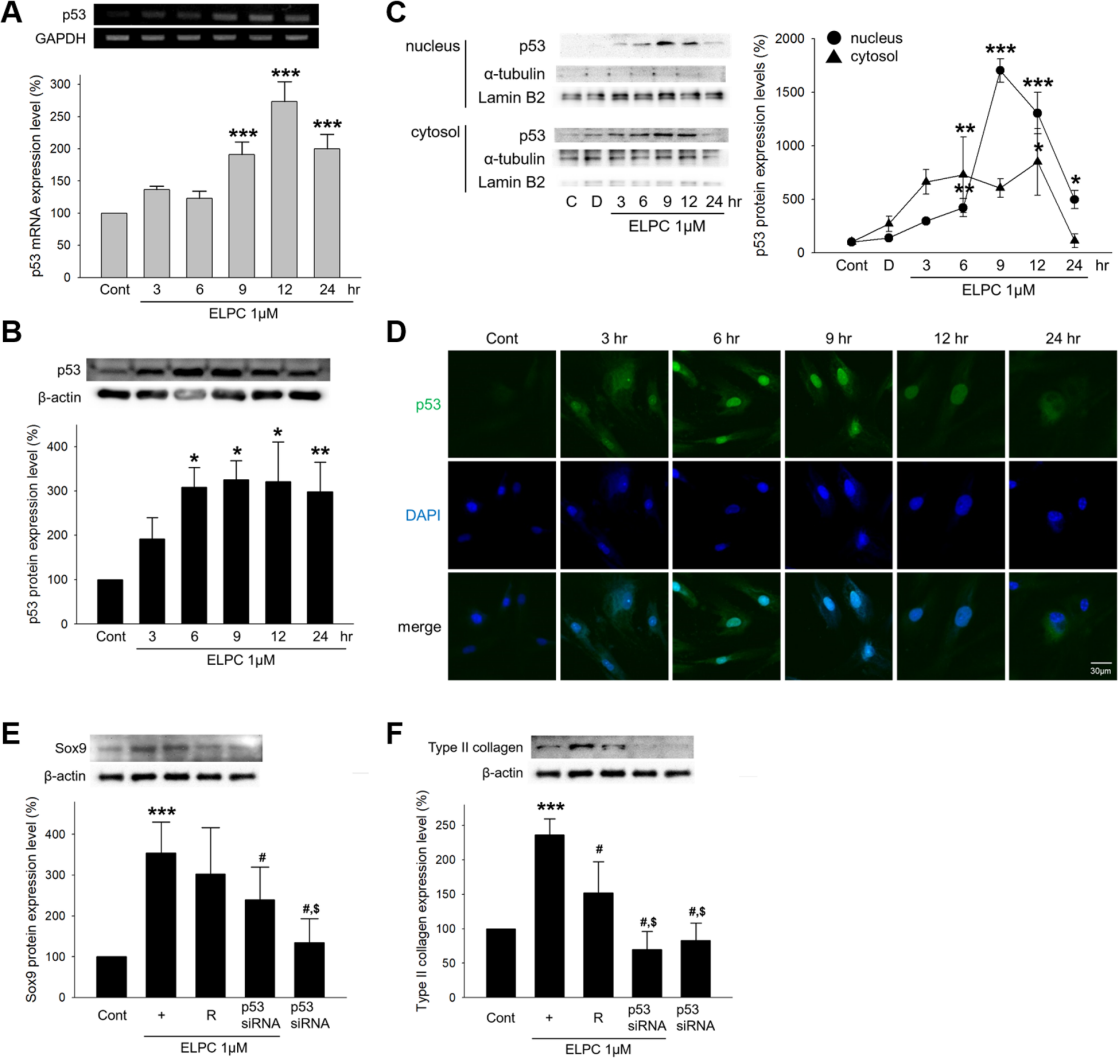
ELPC-induced mechanism in chondrogenic differentiation. **a** The mRNA expression of p53 in the ELPC-treated ASCs. ***p < 0.001 compared to the control. *n* = 3. **b** The protein expression of p53 in the ELPC-treated ASCs. *p < 0.01, **p < 0.05 compared to the control. *n* = 3. **c** The ASCs treated with ELPC for 24 h were separated into the nuclear and cytosolic fractions. p53 was quantified and normalized to Lamin B2 and α-tubulin in the nucleus and cytosol, respectively. Cont Control, D DMSO-treated ASCs. *p < 0.01 **p < 0.05 ***p < 0.001 compared to the control. *n* = 3. **d** The translocation of p53 was confirmed by immunofluorescence staining. p53 was detected by FITC green fluorescence. The nucleus was stained with DAPI (blue). Scale bar: 30 μm. **e**, **f** The protein expression levels of Sox9 and Type II collagen in the ELPC-treated ASCs were investigated 24 h after transfection with 50 nM p53 siRNA. ***p < 0.001 compared to the control, ^#^*p* < 0.05 compared to the ELPC-treated ASCs. ^$^*p* < 0.001 compared to the reagent- and ELPC-treated ASCs. *n* = 3.

We investigated whether ELPC regulates transcription factors that can bind to the promoter region of Sox9. Several transcription factors were selected that were predicted to bind to the Sox9 promoter region by the PROMO 3.0 program. Among them, p53 was found to be associated with ELPC (Fig. 4). Therefore, the mRNA and protein levels of p53 were measured after ELPC treatment. Both the mRNA and protein levels of p53 increased for up to 24 h (Fig. 5a, b). The expression of p53 was also evaluated in isolated nuclear and cytosolic fractions. p53 expression was shifted to the nucleus (Fig. 5c), and a fluorescent stain of p53 also showed translocation from the cytoplasm to the nucleus following ELPC treatment (Fig. 5d). We further confirmed the association between ELPC and p53. The expression of p53 was decreased by p53 siRNA (Fig. S1), which inhibited the expression of Sox9 and Type II collagen (Fig. 5e, f). In addition to p53, we also investigated whether AP-2α or C/EBPβ siRNA regulated the expression of Sox9, resulting in no reduction in mature chondrocyte markers (data not shown). These results indicate that ELPC induces Sox9 expression by regulating the expression of p53 during the chondrogenesis of ASCs.

### Protective effects of ELPC in an animal model of osteoarthritis

**Fig. 6:**
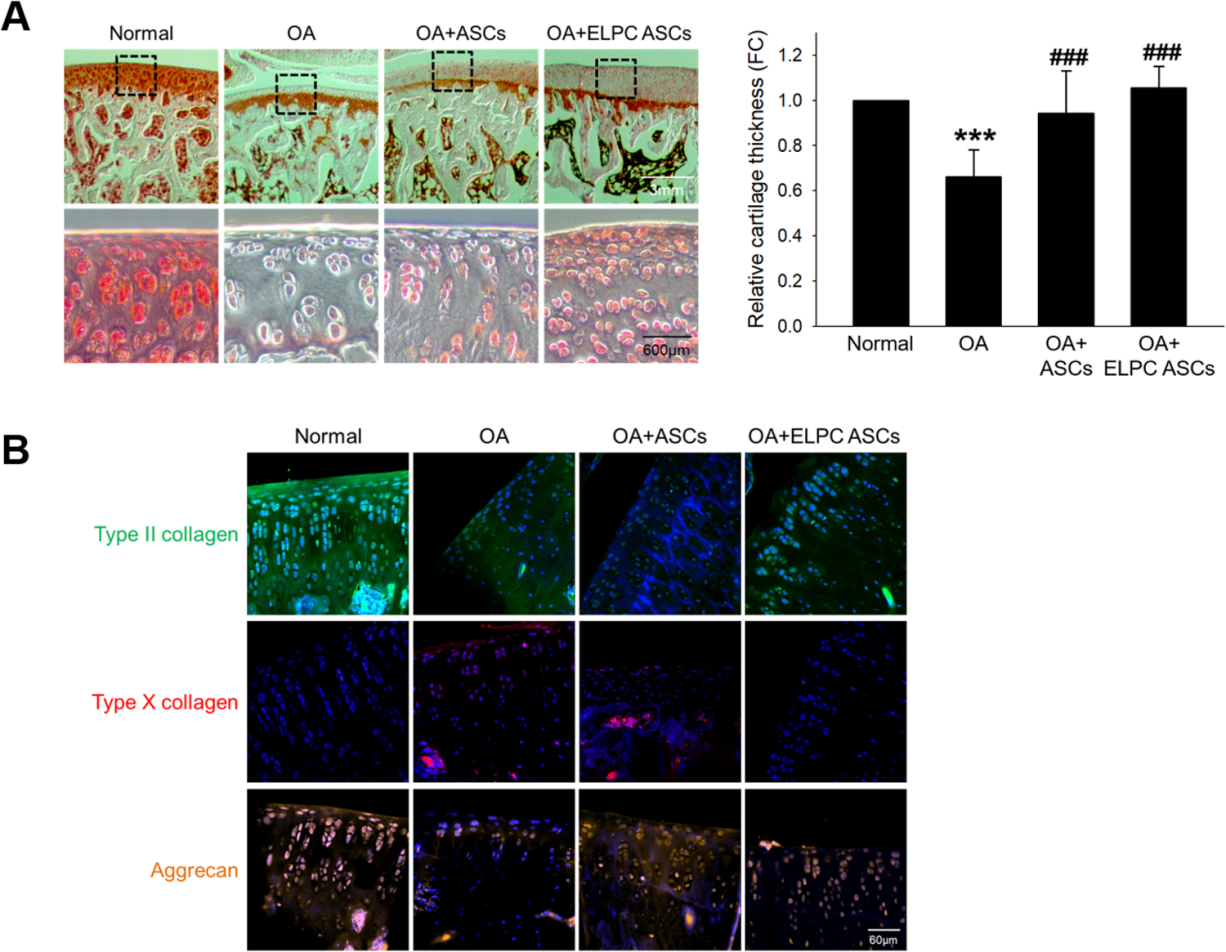
ELPC-induced mechanism and protective effect in an animal model of OA. **a** Safranin O staining of the paraffin sections of the OA animal model. Representative images show cartilage damage after 6 weeks of OA induction. Safranin O binds to glycosaminoglycan in cartilage and is shown in red. The lower figure shows an enlargement of the cartilage part in the upper figure, scale bar: upper 3 mm and lower 600 μm. Graph indicating the relative cartilage thickness. ***p < 0.001 compared to the normal control, ^###^*p* < 0.001 compared to the animal model of OA. *n* = 3. **b** Detection of mature and hypertrophic chondrocyte markers in the OA animal model. Type II collagen (FITC, green), aggrecan (Texas red, red), and type X collagen (rhodamine, orange) expression was measured by immunofluorescence staining and visualized with a confocal microscope. Each section was costained with DAPI (blue) for nuclear staining. The images in the same region of tissue were merged into the overlapped image. Scale bar: 60 μm.

A cartilage-protective effect of ELPC-treated ASCs was observed in an animal model of OA. Decreased cartilage damage was observed in the ASC- and ELPC-treated ASC-injected groups (Fig. 6a). The expression levels of both Type II collagen and Aggrecan were higher than those of the OA-induced group, while the expression level of Type X collagen was lower in the ELPC-treated ASC-injected group than in the ASC-injected group (Fig. 6b).

## Discussion

Our results suggest that ELPC can induce ASC differentiation into chondrocytes through upregulation of the expression of Sox9 by increasing the expression and activation of p53. Most importantly, ASCs differentiated by ELPC did not exhibit the characteristics of hypertrophic chondrocytes. In these experiments, we used the Sox9 promoter-GFP vector system to screen small molecules because Sox9 has a critical role as a transcription factor during cartilaginous skeleton formation in the early stage of chondrogenesis^21–23^ and regulates chondrocyte-specific matrix proteins such as Type II collagen and Aggrecan^24,25^. Therefore, we finally chose ELPC, an alkaloid that was first extracted from the species *Ochrosia elliptica* and *Rauvolfia sandwicensis*. In cancer, ELPC has been used as a drug to induce cell death because it inhibits the enzyme topoisomerase II via intercalative binding to DNA^26^. Therefore, we assessed cell viability and found there was no toxicity until 0.5 μM. In this study, Sox9 overexpression by ELPC treatment was critical, and p53 was shown to play an important role in regulating Sox9 expression. Consequently, ELPC increased the expression and nuclear translocation of p53. Most previous studies of ELPC have explored its effects on various cancers. ELPC inhibited p53 ubiquitination and increased the nuclear localization of endogenous p53^4,27^. Another study also demonstrated that ELPC recovered the transcriptional function of mutant p53^27^. Recent studies have confirmed that p53 regulates the differentiation of embryonic stem cells and induces pluripotent stem cells in response to genomic damage, similar to our results^28,29^.

In vivo studies demonstrated that cartilage damage could be recovered in rats injected with ASCs differentiated by ELPC. In addition, the indicators of mature chondrocytes were increased, and indicators of hypertrophic chondrocytes were not increased (Fig. 6). The limitations of this study include a lack of confirmation of whether differentiated ASCs directly adhere to cartilage to regenerate or are affected by secreted molecules from transplanted ASCs. Therefore, further investigation is needed to confirm these issues. In addition, since there are stem cells in the synovium, it is also necessary to confirm the effect of ELPC treatment alone in amelioration of OA in an in vivo study.

Taken together, the results demonstrate that ELPC induces differentiation into the appropriate type of chondrocytes by increasing Sox9 from ASCs, and ELPC exerted a significant protective effect in an animal model of OA. These results suggest that ELPC is a potential new therapeutic drug for OA.

## Supplementary information

Supporting information
