# Supplementary material for: Differentiation of adipose-derived stem cells into functional chondrocytes by a small molecule that induces Sox9
Source: Exp Mol Med. 2020 Apr 21;52(4):672–81. doi: 10.1038/s12276-020-0424-y (PMC7210883; doi:10.1038/s12276-020-0424-y)
Supplement: Supplementary file 1 — Supporting information [file 12276_2020_424_MOESM1_ESM.docx]

**Differentiation of adipose-derived stem cells into functional chondrocytes by Sox9-induced small molecule**

Jiyun Lee^1,2^, Chang Youn Lee^3^, Jun-Hee Park^3^, Hyang-Hee Seo^1^, Sunhye Shin^3^, Byeong-Wook Song^2^, Il-Kwon Kim^2^, Sang Woo Kim^2^, Seahyoung Lee^2^, Jong-Chul Park^1,4^, Soyeon Lim^2,^*, Ki-Chul Hwang^2,^*

^1^Brain Korea 21 PLUS Project for Medical Science, Yonsei University, Seoul, Republic of Korea

^2^Institute for Bio-Medical Convergence, College of Medicine, Catholic Kwandong University, Gangneung, Republic of Korea

^3^Department of Integrated Omics for Biomedical Sciences, Yonsei University, Seoul, Republic of Korea.

^4^Department of Medical Engineering, Yonsei University College of Medicine, Seoul 03722, Republic of Korea

• Corresponding author: Soyeon Lim, Institute for Bio-Medical Convergence, College of Medicine, Catholic Kwandong University, Gangneung, Republic of Korea. <Tel:82-32-290-2777>, E-mail: slim724@cku.ac.kr

• Corresponding author: Ki-Chul Hwang, Institute for Bio-Medical Convergence, College of Medicine, Catholic Kwandong University, Gangneung, Republic of Korea. Tel: 82-32-290-2773, E-mail: kchwang@cku.ac.kr

**Supporting information**

**Supplementary Figure**


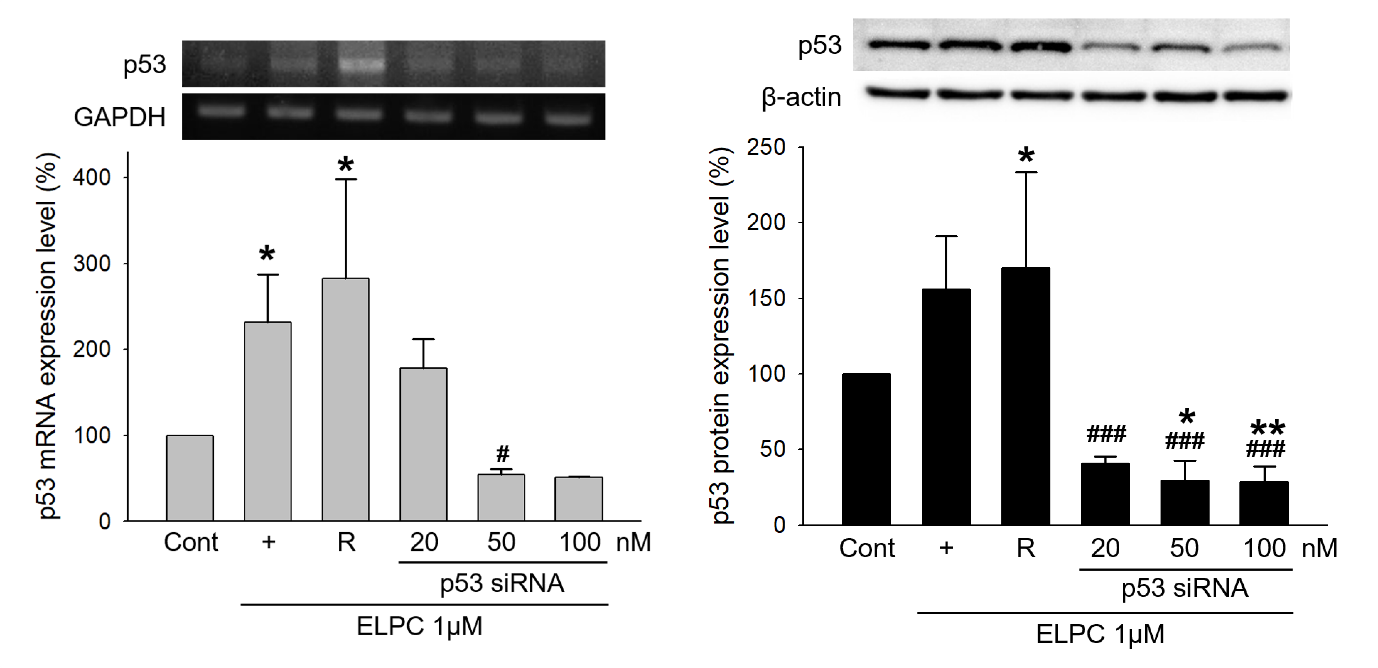


**Supplementary Figure 1.** mRNA and protein expression level of p53 in ASCs which were transfected with p53 siRNA for 24 hr and then treated with ELPC during 12 hr. R: Transfection reagent.

*p<0.05 **p<0.01 compared to Cont, #p<0.05, ###p<0.001 compared to ELPC and ELPC+R. n=3
